# Investigation of time profile of FEV_1_ across the onset of potential COPD: a retrospective cohort study using medical checkup data in Japan

**DOI:** 10.1038/s41598-023-32205-3

**Published:** 2023-04-03

**Authors:** Masaru Suzuki, Isao Matsumoto, Masato Ishida, Yoshiharu Horie, Hideyuki Ban, Wataru Takeuchi, Shunki Nakagawa, Tohru Nakagawa, Tetsuhisa Kitamura, Shigeo Muro

**Affiliations:** 1 Department of Respiratory Medicine, Faculty of Medicine, Hokkaido University, Sapporo, Japan; 2 Department of Respiratory, Inflammation, and Autoimmune, Medical, AstraZeneca K.K., Osaka, Japan; 3 Department of Data Science, Medical, AstraZeneca K.K., Osaka, Japan; 4 Healthcare IT Research Department, Center for Digital Services-Healthcare, Hitachi, Ltd. Research and Development Group, Tokyo, Japan; 5 Hitachi Health Care Center, Hitachi, Ltd., Ibaraki, Japan; 6 Division of Environmental Medicine and Population Sciences, Department of Social and Environmental Medicine, Graduate School of Medicine, Osaka University, Osaka, Japan; 7 Department of Respiratory Medicine, Nara Medical University, Nara, Japan

**Keywords:** Chronic obstructive pulmonary disease, Medical research, Respiratory signs and symptoms

## Abstract

This study compared the time profile of FEV_1_ after COPD diagnosis among rapid decliners, slow decliners, and sustainers in the year of COPD diagnosis. COPD subjects were identified from the annual medical checkup records of Hitachi, Ltd., employees in Japan (April 1998-March 2019). Subjects were categorized into 3 groups (rapid decliner [decrease of FEV_1_ ≥ 63 mL/year], slow decliner [< 63 and ≥ 31 mL/year], and sustainer [< 31 mL/year]) for 5 years. The time profile of FEV_1_ was compared using mixed-effects model for 5 years after diagnosis; risk factors of rapid decliner were detected using logistic model/gradient boosting decision tree. Of 1294 eligible subjects, 18.6%, 25.7%, and 55.7% were classified as rapid decliners, slow decliners, and sustainers, respectively. The annual rates of FEV_1_ decline were similar 3 years before and until COPD diagnosis. The mean FEV_1_ in rapid decliners was 2.82 ± 0.04 L in year 0 and 2.41 ± 0.05 L in year 5, and in sustainers, it was 2.67 ± 0.02 L and 2.72 ± 0.02 L (year 0, p = 0.0004). In conclusion, FEV_1_ declined yearly before diagnosis and the time profiles of FEV_1_ were different in the 3 groups after COPD diagnosis. Therefore, appropriate treatment of the 3 groups with regular lung function tests is necessary to follow FEV_1_ decline after COPD onset.

## Introduction

Chronic obstructive pulmonary disease (COPD) is a leading cause of death globally and a significant driver of healthcare economic costs^1–3^. Irreversible airway obstruction and progressive lung function decline are representative of the nature of the disease^1–4^.

Multiple studies have linked rapid forced expiratory volume in 1 s (FEV_1_) decline with worse disease outcomes^4–9^. A more rapid rate of FEV_1_ decline has been related to more frequent exacerbations^4^. Moreover, rapid FEV_1_ decline is associated with an increased risk of COPD-related hospitalizations and deaths^4–10^. Factors associated with rapid lung function decline include age and sex^11^. A study that included over 10,000 subjects showed that FEV_1_ rapid decliners were older and more likely to be male than nonrapid decliners^11^. In addition, identifying factors associated with rapid FEV_1_ decline at an early stage of COPD is of particular clinical importance because annual FEV_1_ decline is greater at an early stage of COPD than at an advanced stage^12,13^. Several factors are involved in the FEV_1_ decline in COPD subjects, including cigarette smoking and the frequency of exacerbations^4,7^. However, information on risk factors in mild-moderate COPD subjects is limited^14^. Robust real-world evidence is needed to improve our understanding of the nature of rapid FEV_1_ decline.

In the current study, we aimed to compare the time profile of FEV_1_ before and after COPD diagnosis among rapid decliners, slow decliners, and sustainers and identify the patient characteristics by leveraging an annual medical checkup database.

## Methods

### Study design and data source

**Figure 1:**
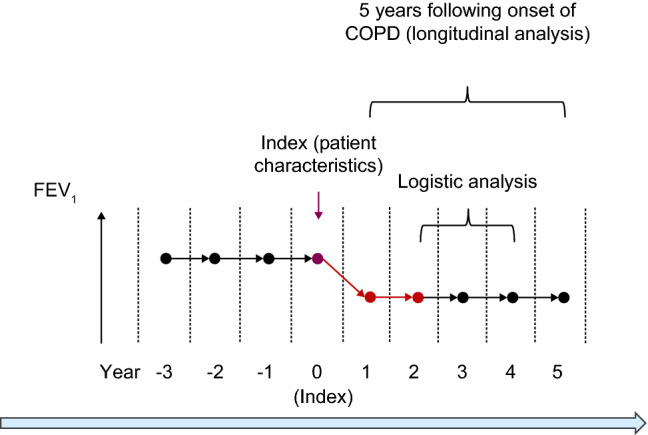
Study design. *COPD* chronic obstructive pulmonary disease, *FEV*_*1*_ forced expiratory volume in 1 s.

This was a retrospective cohort study that analyzed characteristics among subjects with COPD. Data were collected from the annual medical checkup of current and retired Hitachi, Ltd., employees as well as their families in Japan from April 1998 to March 2019. This database includes the data for approximately 16,000 employees and their families, with ages ranging from 18 to 75 years. Details of the data source have been described in our previous study^15^. Subjects with COPD aged 30–75 years who had undergone at least 3 annual medical checkups within 5 years were analyzed for this study. The index date was defined as the date of COPD diagnosis (Fig. 1). The annual medical checkup includes clinical measurements such as routine FEV_1_ in the absence of suspected COPD and questionnaires to examine the health conditions of employees (see Supplementary Table 1). Information on the equipment used is unavailable, and spirometry was conducted in accordance with the guideline recommended by the Japanese Respiratory Society for lung function testing^16^. Individual informed consent was obtained using an opt-out model in agreement with the Institutional Review Board at Hitachi, Ltd. This study was conducted in accordance with the ethical principles of the Declaration of Helsinki.

### Study population

Subjects with a pre-bronchodilator (pre-BD) FEV_1_/forced vital capacity (FVC) measurement of < 0.7 during an annual lung function test for 2 consecutive years were defined as having COPD^17^. Subjects with a pre-BD FEV_1_/FVC measurement of ≥ 0.7 in at least 3 consecutive annual lung function tests were regarded as non-COPD subjects^17^. When more than 3 records were available for non-COPD subjects, the 3 most recent records were analyzed. Individuals with ˂ 2 lung function tests were excluded. Those who had lung cancer or asthma were excluded, as most subjects with asthma-COPD overlap had a confirmed asthma diagnosis, were predominantly female, had a longer duration of COPD, used respiratory medications and statins more frequently, and had more comorbidities, which may introduce a bias^15,18^. In order to compare the time profiles of FEV_1_ over 5 years after COPD diagnosis, subjects were categorized by their FEV_1_ measurements based on the criteria used in the Hokkaido COPD Cohort Study: ≥ 63 mL/year as rapid decliners, < 63 and ≥ 31 mL/year as slow decliners, and < 31 mL/year as sustainers^19^.

### Outcomes

The time profile of FEV_1_ evaluated across COPD diagnosis was the primary outcome of this study. Characteristics of subjects diagnosed with COPD were also compared between the 3 groups. The factors associated with a rapid FEV_1_ decline were investigated using a machine learning (ML) approach between rapid decliners and nonrapid decliners (slow decliners + sustainers). The answers obtained through a questionnaire at the year of COPD diagnosis were summarized.

### Statistical analysis

#### Lung function time profile after COPD diagnosis (longitudinal analysis)

FEV_1_ of the 3 groups was calculated for 5 years after COPD diagnosis. The time profiles of the 3 groups were compared using a mixed-effects model that included baseline value, sex, age (< 60 years, ≥ 60 years), smoking status, body mass index (BMI; < 25 kg/m^2^, ≥ 25 kg/m^2^), and blood eosinophil (EOS) count (< 200 cells/mm^3^, ≥ 200 cells/mm^3^) as fixed effects, and time as a random variable.

#### Detection of factors associated with rapid decliners

Logistic regression and gradient boosting decision tree (XGBoost) were applied to predict the factors associated with rapid FEV_1_ decline, as described in our previous report^15^. Models were constructed in the training sets and validated for model evaluation in the test datasets. The training dataset that consisted of eligible subjects was first generated and then randomly split into 80% for model construction and the remaining 20% for evaluation of model performance. A test dataset was also created. The ratio of training and test sets was 4:1. The process from data split to model evaluation was repeated 5 times for cross-validation (fivefold cross-validation). The most fitted model was investigated by area under the curve (AUC). The contributions of each predictor to the constructed model were examined by calculating the feature importance. Statistical analyses were performed using R version 3.6 (R foundation for Statistical Software) and Python 3.6 (Python Software Foundation). p values are shown in all comparisons, and p-value that shows less than 5% was considered a statistically significant difference.

### Ethics approval and consent to participate

The study protocol was reviewed and approved by the ethics committee of MINS (a non-profit organization in Tokyo, Japan) and the Research and Development Group and Corporate Hospital Group of Hitachi, Ltd. (Tokyo, Japan) prior to the start of data analysis. Individual informed consent was obtained using an opt-out model in agreement with the Institutional Review Board at Hitachi, Ltd. This study was conducted in accordance with the ethical principles of the Declaration of Helsinki.

## Results

### Patient disposition

**Figure 2:**
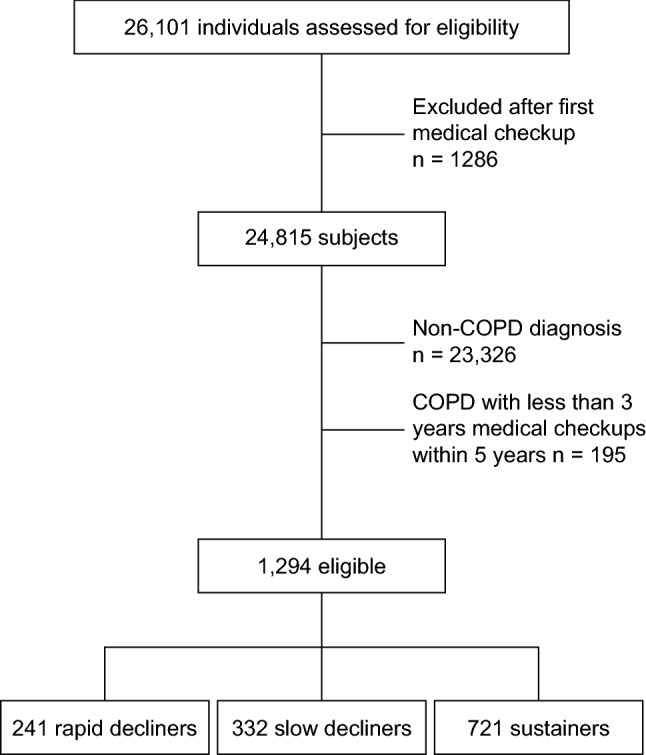
Patient disposition.

Of 26,101 subjects, 24,807 were excluded (Fig. 2) as they met the exclusion criteria. In total, 1294 were included in the study; 241 (18.6%) were classified as rapid decliners, 332 (25.7%) as slow decliners, and 721 (55.7%) as sustainers.

### Characteristics of subjects diagnosed with COPD in each group

Patient characteristics are shown in Table 1. Fewer current smokers were observed in the sustainers than in the rapid or slow decliners. There were more ex-smokers and nonsmokers in the sustainers compared with the others. Lower means of BMI (22.6 kg/m^2^ vs 23.05 kg/m^2^), waist circumference (80.9 cm vs 82.5 cm), and body fat percentage (20.2% vs 21.5%) were seen in the rapid decliners compared with the sustainers group (Table 1).

**Table 1:** Patient characteristics.

	Rapid decliners	Slow decliners	Sustainers
N	241	332	721
Age, years	55.4 (8.7)	56.4 (8.8)	55.7 (9.2)
Female, n (%)	7 (2.9)	14 (4.2)	29 (4.0)
Smoking status, n (%)	*		
Current	153 (63.5)	184 (55.4)	365 (50.6)
Ex	50 (20.7)	103 (31.0)	219 (30.4)
Never	37 (15.4)	45 (13.6)	136 (18.9)
BMI, kg/m^2^	22.6 (2.7)*	22.5 (2.4)*	23.05 (2.7)
Body fat, %	20.3 (4.5)*	20.8 (4.4)*	21.5 (4.6)
Waist circumference, cm	80.9 (7.4)*	81.9 (7.5)	82.5 (7.4)
HbA1c, %	5.4 (0.6)*	5.5 (0.8)	5.5 (0.7)
LDL cholesterol, mg/dL	115.4 (30.7)*	117.8 (29.3)	119.9 (28.9)
MCH, pg	31.8 (1.7)*	31.5 (1.8)*	31.3 (1.7)
MCHC, g/L	33.8 (0.8)*	33.7 (0.8)*	33.6 (0.8)
MCV, fL	93.8 (4.5)*	93.4 (4.7)	93 (4.4)
RBC, × 10^4^/µL	471.1 (37.5)*	472.6 (38.9)	477.1 (38.5)
CRE, mg/dL	0.8 (0.1)*	0.8 (0.1)*	0.8 (0.1)
TC, mg/dL	194.2 (30.5)*	195.6 (31.7)	199.2 (31.3)
ALB, g/dL	4.2 (0.3)*	4.2 (0.3)*	4.3 (0.3)
EOS, %	3.3 (2.2)*	3.3 (1.9)	3.6 (2.2)
eGFR, mL/min/1.73 m^2^	80.7 (14.3)*	79.7 (15.4)	78.2 (15.6)
EOS, cells/mm^3^	195 (132)*	200 (123)	216.3 (143.3)
LH ratio	2.2 (0.8)*	2.3 (0.9)	2.3 (0.8)
Lung function			
FEV_1_, mL	2.8 (0.6)*	2.7 (0.5)	2.7 (0.5)
FVC, mL	4.2 (0.8)*	4.0 (0.8)	4.0 (0.8)
%VC	110.0 (16.2)*	106.8*	103.8 (15.2)
FEV_1_/FVC, %	67.0 (2.3)	67.1 (2.1)	66.8 (2.7)

*Statistically significant difference vs sustainers at < 0.05 found by the Fisher exact test.

Data are presented as mean (SD) unless stated otherwise.

*ALB* albumin, *BMI* body mass index, *CRE* creatinine, *eGFR* estimated glomerular filtration rate, *EOS* eosinophil, *FEV*_*1*_ forced expiratory volume in 1 s, *FVC* forced vital capacity, *HbA1c* hemoglobin A1c, *LDL* low-density lipoprotein, *LH ratio* LDL/HDL ratio, *MCH* mean corpuscular hemoglobin, *MCHC* mean corpuscular hemoglobin concentration, *MCV* mean corpuscular volume, *N* number of subjects, *RBC* red blood cell, *SD* standard deviation, *TC* total cholesterol, *VC* vital capacity.

Rapid decliners had higher lung function (mean ± standard error) at the year of COPD diagnosis in comparison with sustainers (FEV_1_ = 2.82 ± 0.04 L vs 2.67 ± 0.02 L [p = 0.0004], FVC = 4.18 ± 0.04 L vs 3.93 ± 0.05 L [p = 0.0003], and percentage vital capacity (%VC) = 110% vs 103.8% [p < 0.000001]).

The answers obtained through a questionnaire at the year of COPD diagnosis are summarized in Table 2. A statistically significant difference in sleep duration between rapid decliners and sustainers was observed (p < 0.000001). A total of 21 (8.71%) rapid decliners reported sleeping for over 7 h during the past months vs 113 (15.67%) sustainers. Most rapid decliners slept in a range of 5–6 h during the past month vs 6–7 h reported by sustainers. Regarding the degree of physical activity at work, a significant difference was observed between rapid decliners and sustainers, wherein rapid decliners engaged in increased workplace activity as compared to sustainers. Similarly, decliners’ daily consumption of breakfast (rapid decliners: p < 0.000001 and slow decliners: p = 0.000001) and regular exercise (rapid decliners: p = 0.000018 and slow decliners: p = 0.000002) varied significantly from the sustainers.

**Table 2:** Questionnaire-related outcomes.

	Rapid decliners	Slow decliners	Sustainers
N	241	332	721
Sleep duration in the past month, n (%), h	*		
Missing	91 (37.8)	110 (33.1)	130 (18.0)
< 5 h	10 (4.1)	10 (3.0)	26 (3.6)
> 5–< 6 h	61 (25.3)	67 (20.2)	221 (30.7)
> 6– < 7 h	58 (24.1)	99 (29.8)	231 (32.0)
> 7 h	21 (8.7)	46 (13.9)	113 (15. 7)
Amount of alcohol consumed per day, L, mean (SD)	1.2 (1.1)*	1.2 (1.2)	1.0 (1.0)
Eating breakfast every day, n (%)	*	*	
Missing	95 (39.4)	117 (35.2)	139 (19.3)
Always	106 (44.0)	168 (50.6)	438 (60.7)
Often	14 (5.8)	25 (7.5)	79 (11.0)
Sometimes	17 (7.1)	10 (3.0)	43 (6.0)
Rarely	9 (3.7)	12 (3.6)	22 (3.1)
Walking time during commuting, min, mean (SD)	15.7 (18.5)	16.0 (17.7)	17.3 (19.9)
Degree of physical activity at work, n (%)	*		
Missing	49 (20.3)	94 (28.3)	189 (26.2)
Sedentary work	86 (35.7)	120 (36.1)	306 (42.4)
Standing (light)	29 (12.0)	32 (9.6)	67 (9.3)
Walking (medium)	61 (25.3)	61 (18.4)	120 (16.6)
Heavy work	16 (6.6)	25 (7.5)	39 (5.4)
Regular exercise, n (%)	*	122 (36.74)*	162 (22.46)
Missing	1 (0.4)		0
Yes	152 (63.1)		559 (77.5)
No	88 (36.5)		162 (22.5)

*Statistically significant difference vs sustainers at < 0.05.

*N* subjects, *SD* standard deviation.

### Lung function trajectories across the diagnosis of COPD

**Figure 3:**
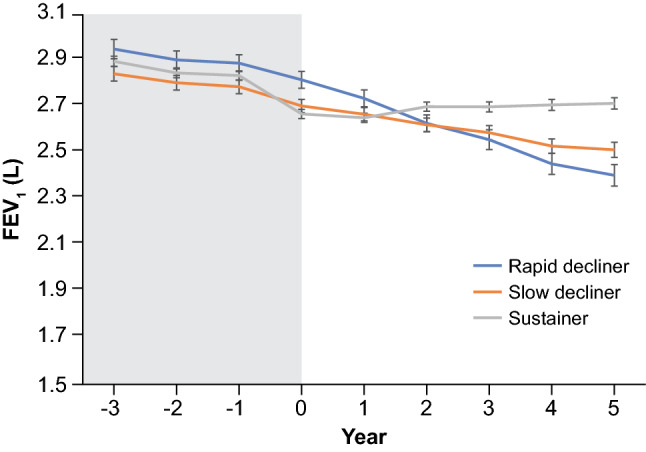
Time profile of lung function across the diagnosis of COPD. *FEV*_*1*_ forced expiratory volume in 1 s, *L* liter.

The time profile of lung function across the diagnosis of COPD is shown in Fig. 3. The time profile in rapid decliners was statistically different from that in slow decliners or sustainers (p = 0.0001).

Rapid decliners showed the highest mean ± standard error FEV_1_ of 2.82 ± 0.04 L in year 0 among the 3 groups; this mean value sharply declined to 2.41 ± 0.05 L in year 5. Slow decliners presented a mean FEV_1_ of 2.71 ± 0.03 L in year 0, which steadily declined to 2.52 ± 0.03 L in year 5. The mean FEV_1_ of sustainers was 2.67 ± 0.02 L in year 0, which was slightly lower compared with that of slow decliners, increased to 2.70 ± 0.02 L in year 2, and then continued to increase to 2.72 ± 0.02 L in year 5. The time profile of FEV_1_ was created for 3 years prior to the onset of COPD (Fig. 1). Annual rates for the decline were similar from year − 3 to year 0, except for sustainers with the highest mean FEV_1_ decline at index (− 0.07 mL). There was a large decline in sustainers from year − 1 to year 0.

### Factors associated with a rapid decline in FEV_1_

The gradient boosting decision tree model (AUC = 0.516) identified factors associated with a rapid FEV_1_ decline that showed higher importance (see Supplementary Fig. S1 including the top 30 factors) and those based on answers to questionnaires (see Supplementary Fig. S2). Mean FVC decline has been presented in Supplementary Fig. S3. The results of the logistic regression model indicated that vital capacity, mean corpuscular hemoglobin, smoking status, cough, platelet count, total protein, HbA1c, FEV_1_% (see Supplementary Fig. S4), and uric acid were predictive of rapid decliners. In contrast, arrhythmia, other diseases, FEV_1_, mean corpuscular volume, BMI, mean corpuscular hemoglobin concentration, albumin, C reactive protein, smoking duration, hematocrit, aspartate aminotransferase, percentage VC, and duration of smoking cessation were unlikely to predict rapid decliners (see Supplementary Table 2). Generally consistent with these results were those obtained using a logistic regression model, including questionnaire-obtained data comprising lifestyle, symptoms, and treatment-related information (see Supplementary Table 3).

## Discussion

Studies have reported the rate of FEV_1_ decline among the total or male Japanese population^20,21^. In contrast, our study evaluated the time profile of FEV_1_ capacity grouped by 3 categories (rapid decliners, slow decliners, and sustainers) using datasets, which included working-age Japanese adults, with information collected for over 20 years, providing further characterization for the time profile of FEV_1_ among subjects with varying rates of decline over time, consistently measured over the course of 8 years.

In the current study, the proportion of rapid decliners among COPD subjects, particularly those newly diagnosed with COPD, remains largely unclear, and 241 (18.6%) were classified according to the Hokkaido cohort as rapid decliners^19^. Subjects with a higher basal FEV_1_ showed a steeper decline in lung function, possibly because early-stage COPD subjects with preserved FEV_1_ might “have more lung function to lose” than those with a more advanced stage of COPD^22^. Although some similarities were noted, the declines in FEV_1_ were not exactly parallel among the three groups; for rapid decliners, the greatest decline in lung function was observed during the second year of follow-up, reflecting the accelerated loss of lung function usually observed in the early stage of COPD^14,22^. The onset of COPD was unpredictable based on the level of annual FEV_1_ decline between year − 3 and year 0, which were similar between all 3 groups except for the sharp decline in the sustainers between year − 1 and 0. Since no relevant difference was observed in the annual FEV_1_ decline among the 3 groups, the onset of COPD cannot be determined based on this factor; thus, regular lung function tests are necessary to detect the onset of COPD in a timely manner.

In our study, current smokers were more prevalently rapid decliners and slow decliners compared with sustainers. Although the ML model had a low AUC, it was able to identify smoking status, pack-year, smoking duration, and duration of smoking cessation as potential risk factors for a rapid FEV_1_ decline. Multiple studies have validated cigarette smoking as a risk factor for accelerated lung function decline^7,20,23,24^. Smoking cessation may reverse or alleviate the damage incurred over time and restore lung function to near-normal FEV_1_ values^7,20,23^.

BMI and body fat percentage were lower in rapid decliners compared with sustainers at the diagnosis of COPD. Similar to previous reports, our ML results support an inverse relationship between BMI and a rapid FEV_1_ decline^25–27^. This finding has been described as the “obesity paradox” in COPD; a high BMI shows a protective effect, whereas a low BMI has been identified as a factor associated with accelerated lung function decline^28^.

A rapid FEV_1_ decline was associated with several lifestyle-related factors. The characteristics describing rapid decliners were short sleeping hours, omission of breakfast, increased alcohol consumption, lack of regular exercise, and high level of physical activity at work. Of note, ML results supported these findings, except for omission of breakfast which showed an opposite trend in ML. Although the discrepancy between patient characteristics and ML findings remains unknown, our findings possibly indicate a role of frequent activity, adequate sleep, and moderate alcohol consumption in preventing a rapid FEV_1_ decline.

Our analysis has several limitations. First, complete medical and medication history of the participants was not available, which may have impacted the degree of COPD progression; nevertheless, pre-BD lung test was conducted as part of annual medical health checkup. The impact of longitudinal changes in lifestyle on disease trajectory remains a limitation of database research. The reasons for missing data vary broadly, including unrecorded collection during an office visit^29,30^. Hence, our collected data might not necessarily fully encompass the natural clinical course. For example, the potential contributions of specific comorbidities in the decline in lung function could not be established. Second, using a single database may give biased results, such as overlooking the influence of occupational exposure and the development of COPD^31,32^. Consequently, to generalize these results and obtain a broader vision of the general population, it is necessary to include multiple databases, especially those from other countries; thus, further studies will be required to evaluate the factors associated with a rapid FEV_1_ decline in subjects at risk of developing COPD across a diverse population. Although sensitivity analysis based on FEV_1_ percentile showed similar results (data not shown), we were not able to categorize patients into the 3 groups based on age-matched lower-limit of normality values using this data. Finally, we obtained our results based on the available pre-BD FEV_1_ values in the absence of post-BD FEV_1_ values. However, COPD prevalence significantly differs when COPD was diagnosed based on pre-BD compared with post-BD spirometry measurements^33^. Biological variability and/or measurement errors can give rise to known and expected variations in spirometry values upon repeated testing^34,35^. Decline in FEV_1_ values can also be seen in cardiovascular and respiratory disorders^36^. Although airflow limitation (pre-BD FEV_1_) is not paramount for COPD diagnosis as systemic inflammation and other clinical signs may not be captured by FEV_1_, data suggest a link between airflow limitation and prognosis^34,37^. Nonetheless, pre-BD FEV_1_ values are known to possibly overestimate COPD prevalence through high false-positive rates; thus, post-BD FEV_1_ values are usually a more accurate predictor of COPD outcomes, and the data here need to be interpreted with caution^38–40^. Unfortunately, we could not clarify the importance of “rapid decline of FEV_1_ over time” as a definitive patient characteristic. Ideally, COPD diagnosis requires serial longitudinal spirometry assessments, which should be accompanied by a comprehensive clinical assessment^34^.

## Conclusions

FEV_1_ declined yearly before diagnosis in rapid decliners, slow decliners, and sustainers. The time profiles of FEV_1_ were different in the 3 groups after COPD diagnosis. Therefore, appropriate treatment of the 3 groups with regular lung function tests is necessary to follow the FEV_1_ decline after COPD onset in a timely manner.

## Supplementary Information

Supplementary Information.

## Data Availability

The datasets generated and/or analyzed during the current study are not publicly available but are available from the corresponding author on reasonable request.

## Abbreviations

AUC: Area under the curve
BMI: Body mass index
COPD: Chronic obstructive pulmonary disease
EOS: Eosinophil
FEV_1_: Forced expiratory volume in 1 s
FVC: Forced vital capacity
ML: Machine learning
pre-BD: Pre-bronchodilator
VC: Vital capacity
