# Supplementary material for: Investigation of time profile of FEV1 across the onset of potential COPD: a retrospective cohort study using medical checkup data in Japan
Source: Sci Rep. 2023 Apr 3;13:5454. doi: 10.1038/s41598-023-32205-3 (PMC10070435; doi:10.1038/s41598-023-32205-3)
Supplement: Supplementary file 1 — Supplementary Information. [file 41598_2023_32205_MOESM1_ESM.docx]

**Supporting information**

**METHODS**

***Outcome measures***

The data source was annual medical check-up data for all Hitachi employees from April 1998 to March 2019. Data were archived in a high-security server that was managed with limited access rights by Hitachi [1]. The annual medical check-up includes clinical measurements and questionnaires to examine the health of employees (Supplementary Table 1). Such questionnaires are utilized by Japanese organizations to evaluate their employees’ health and give advice about health promotion, such as giving up smoking and exercising regularly based on the second term of the National Health Promotion Movement in the 21st century (Health Japan 21) issued by the Ministry of Health, Labour, and Welfare in Japan [2]. Spirometry was calibrated and performed by trained paramedical personnel according to the American Thoracic Society/European Respiratory Society guidelines [3, 4].

**Supplementary Fig. S1:** Predictors for developing rapid FEV_1_ decline in 3 years.


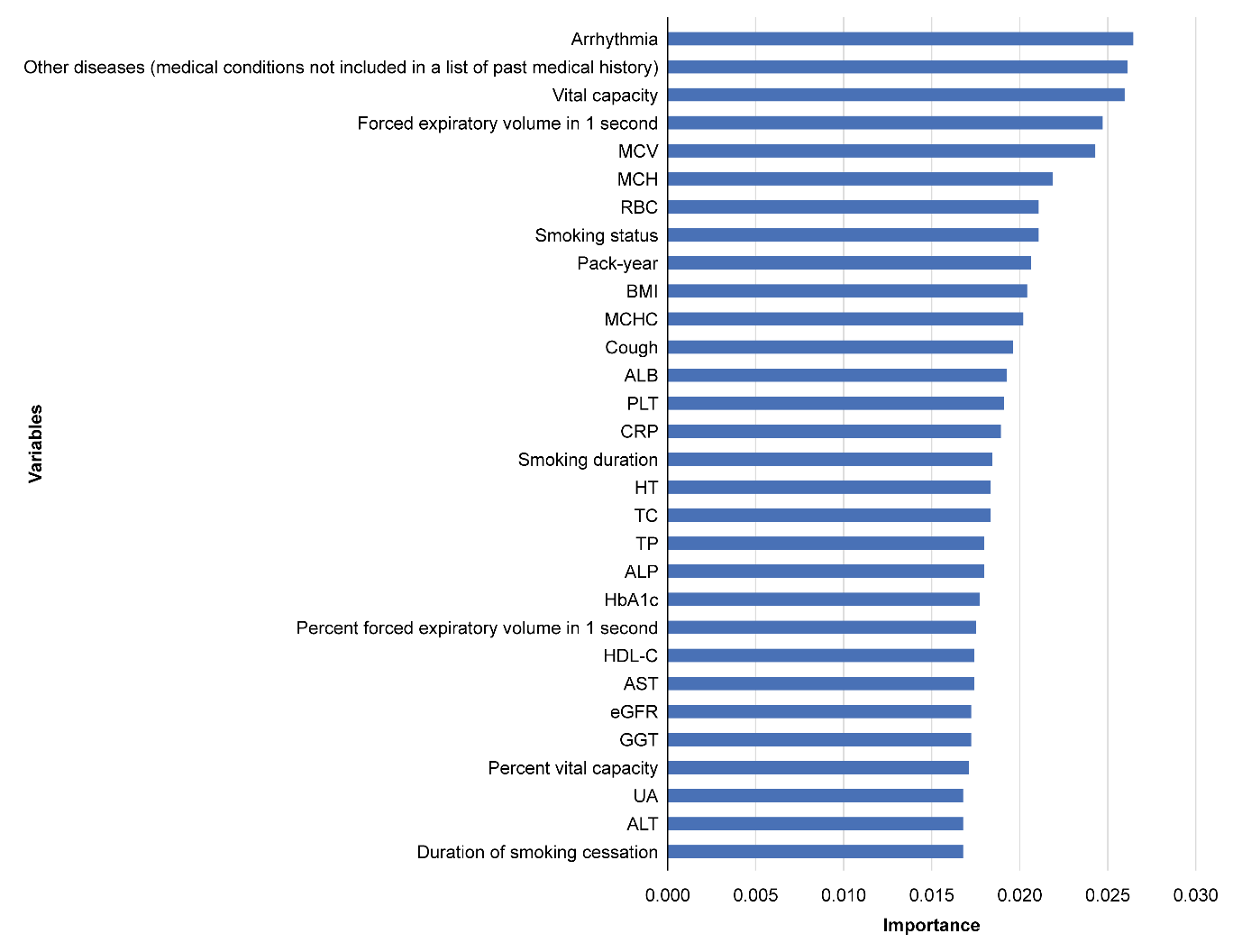


**Supplementary Fig. S2:** Predictors including questionnaire data for developing rapid FEV_1_ decline in 3 years

**
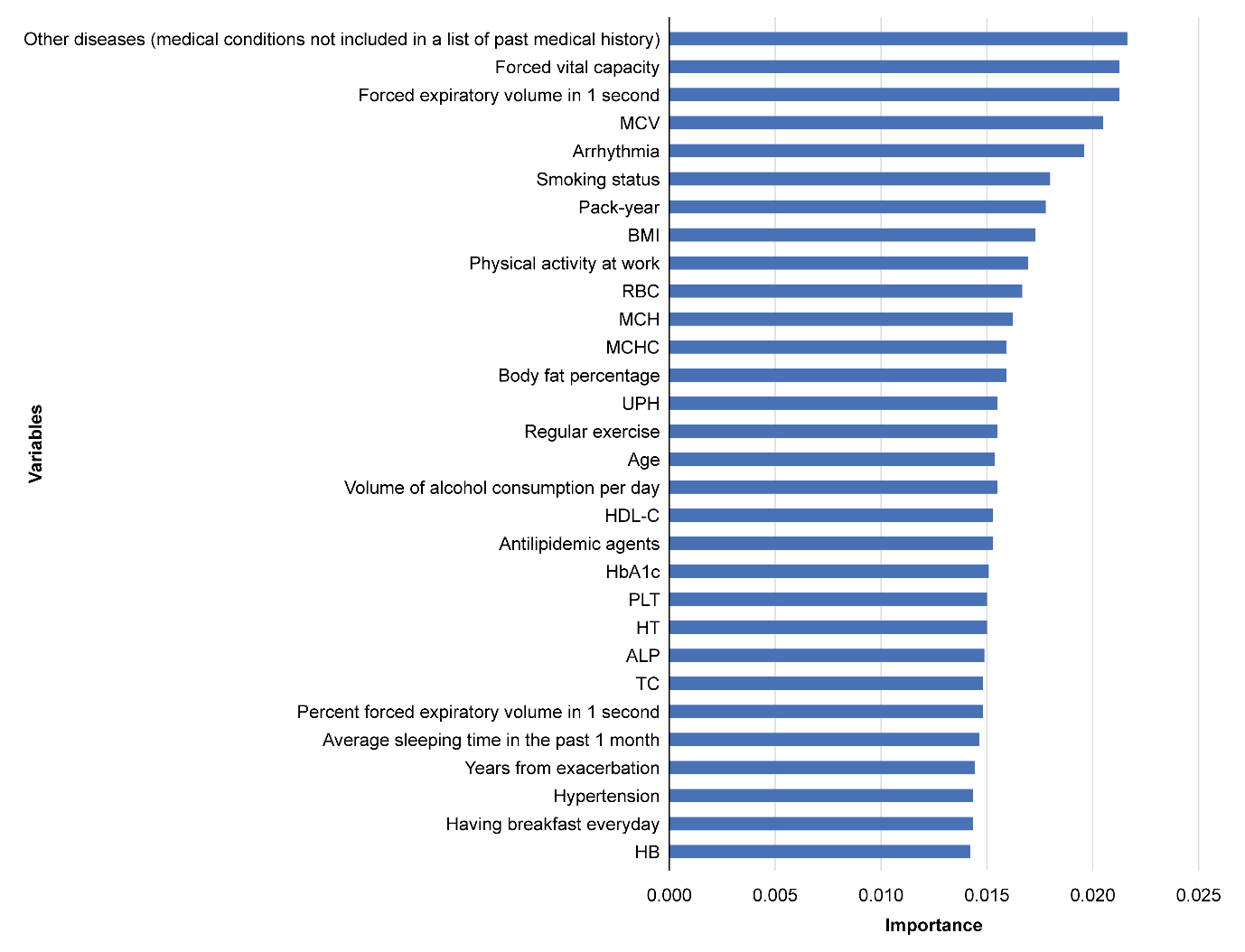
**

**Supplementary Fig. S3:** Time Profile of FVC across the diagnosis of COPD


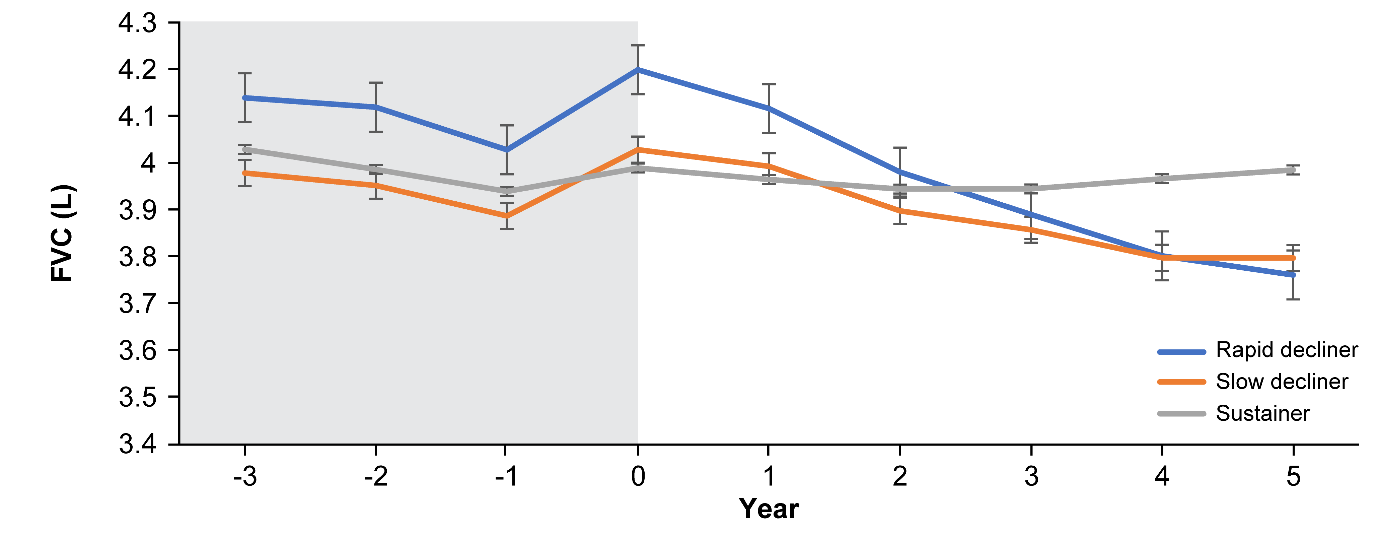


Data are presented as mean (SE)

COPD, chronic obstructive pulmonary disease; FVC, forced vital capacity; SE, standard error

**Supplementary Fig. S4:** Time profile of FEV_1_% across the diagnosis of COPD


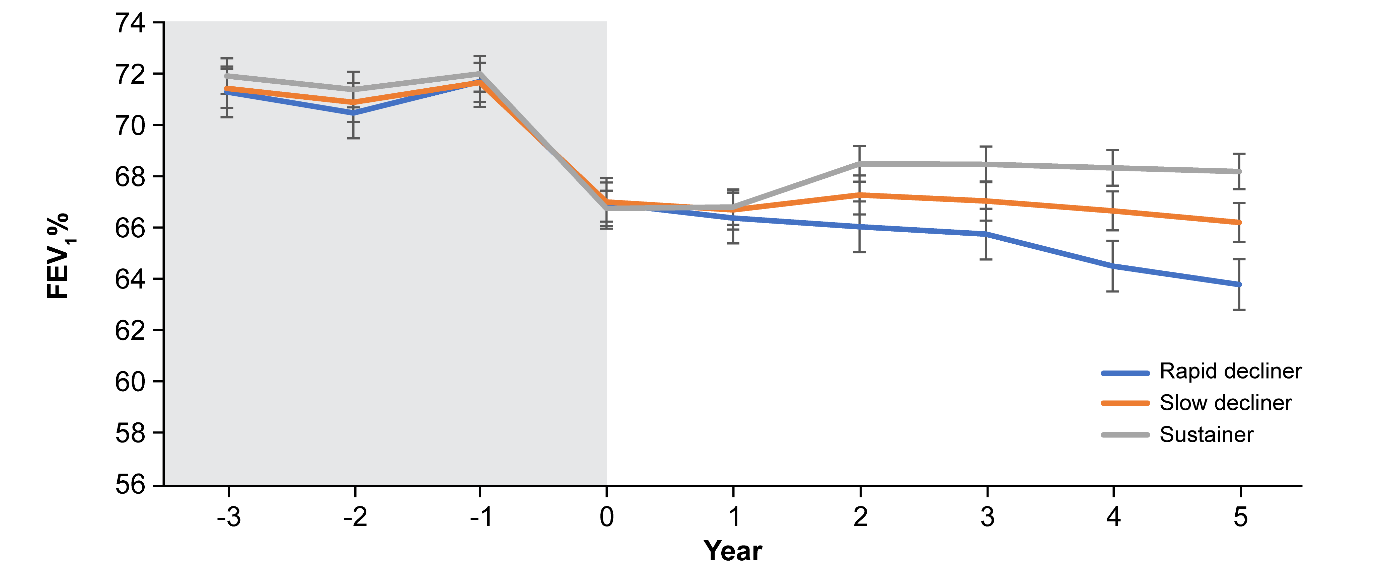


Data are presented as mean (SE)

COPD, chronic obstructive pulmonary disease; FEV_1_, forced expiratory volume in 1 second; FVC, forced vital capacity; SE, standard error

**References**

1. Muro, S. *et al*. Machine learning methods for the diagnosis of chronic obstructive pulmonary disease in healthy subjects: retrospective observational cohort study. *JMIR Med. Inform*. **9,** e24796 (2021).
2. Sugiyama, K. *et al*. Awareness and health consciousness regarding the national health plan “Health Japan 21” (2nd edition) among the Japanese population in 2013 and 2014. *Nihon Koshu Eisei Zasshi*. **63,** 424–431.
3. Miller, M. R. *et al*. Standardisation of spirometry “ATS/ERS Task Force: Standardisation of Lung Function Testing”. *Eur. Respir. J*. **26,** 319–338 (2005).
4. Celli, B. R., MacNee, W. & ATS/ERS Task Force. Standards for the diagnosis and treatment of patients with COPD: a summary of the ATS/ERS position paper. *Eur. Respir. J*. **23,** 932–946 (2004).

**Supplementary Table 1**. Questionnaire items

| **Type** | **Measurement and Unit** |
| --- | --- |
|  |  |
| Demographic | Smoking status, n |
|  | Sex, n |
|  | Age, years |
| Lung function test | FEV_1_, L |
|  | VC, L |
|  | FEV_1_/FVC, % |
|  | %VC, % |
|  | %FEV_1_, % |
| Vitals | BMI, kg/m^2^ |
|  | Body fat ratio, % |
|  | Diastolic blood pressure, mmHg |
|  | Systolic blood pressure, mmHg |
| Laboratory tests | ALP, U/L |
|  | BUN, mg/dL |
|  | AST, U/L |
|  | ALT, U/L |
|  | HbA1c, % |
|  | HDL-C, mg/dL |
|  | Hematocrit, % |
|  | Hb, g/dL |
|  | KET, n |
|  | LDL, mg/dL |
|  | MCH, pg |
|  | MCHC, g/L |
|  | MCV, fL |
|  | PLT, ×10^4^/µL |
|  | RBC, ×10^4^/µL |
|  | CRE, mg/dL |
|  | T-BIL, mg/dL |
|  | TC, mg/dL |
|  | Fasting blood sugar, mg/dL |
|  | TP, g/dL |
|  | U-CRE, mg/day |
|  | U-RBC, n |
|  | UA, mg/dL |
|  | Protein in urine, n |
|  | Urinary sugar, n |
|  | UPH, pH |
|  | U-WBC, n |
|  | WBC, ×10^2^ /µL |
|  | GGT, U/L |
|  | ALB, g/dL |
|  | CHE, U/L |
|  | Occult blood in urine, n |
|  | D-BIL, mg/dL |
|  | EOS, % |
|  | IRI, µU/mL |
|  | Urobilinogen in urine, n |
|  | SG, ratio |
|  | Pulse, times/min |
|  | CRP, mg/dL |
|  | eGFR, mL/min/1.73m^2^ |
|  | EOS_number, /mm^3^ |
| Questions about lifestyle habits | Average sleeping time in the past 1 month, hour |
|  | Number of alcohol consumption days per week, n |
|  | Amount of alcohol consumed per day, L |
|  | “I have breakfast everyday”, n |
|  | Walking time for commuting to work, hour |
|  | Physical activity at work, hour |
|  | Regular exercise, hour |
| Questions about symptom | “I have palpitation or disturbed pulse”, n |
|  | “I have chest compression and pain”, n |
|  | “I have cough and sputum”, n |
|  | “I have gastric distress and nausea”, n |
|  | “I have stiff neck or back pain”, n |
|  | “My fingers or arms are numb or painful”, n |
|  | “I have low back pain”, n |
|  | “My hip, thighs, or calf are numb or painful”, n |
| Questions about treatment history | “I take anti-hypertension drugs”, n |
|  | “I take insulin injection or anti-hyperglycemic drugs”, n |
|  | “I take anti-hyperlipidemic drugs”, n |
|  | “I am under treatment_other diseases”, n |
|  | “I am under treatment_epilepsy”, n |
|  | “I am under treatment_allergic diseases”, n |
|  | “I am under treatment_arrythmia”, n |
|  | “I am under treatment_insomnia”, n |
|  | “I am under treatment_duodenal ulcer”, n |
|  | “I am under treatment_colorectal polyp”, n |
|  | “I am under treatment_myocadiac infarction”, n |
|  | “I am under treatment_malignancy”, n |
|  | “I am under treatment_chronic hepatitis”, n |
|  | “I am under treatment_slipped disk”, n |
|  | “I am under treatment_bronchial asthma”, n |
|  | “I am under treatment_angina”, n |
|  | “I am under treatment_throid diseases”, n |
|  | “I am under treatment_psychiatric diseases”, n |
|  | “I am under treatment_diabetes”, n |
|  | “I am under treatment_tuberculosis”, n |
|  | “I am under treatment_stomach ulcer”, n |
|  | “I am under treatment_gallstone”, n |
|  | “I am under treatment_liver steatosis”, n |
|  | “I am under treatment_stroke”, n |
|  | “I am under treatment_kidney diseases”, n |
|  | “I am under treatment_anemia”, n |
|  | “I am under treatment_rheumatoid arthritis, collagen diseases”, n |
|  | “I am under treatment_hyperuricemia”, n |
|  | “I am under treatment_hyperlipidemia”, n |
|  | “I am under treatment_hypertension”, n |
|  | “I had surgery_lung”, n |
| Electrocardiogram (ECG) | ECG_trigeminal pulse, n |
|  | ECG_bigeminal pulse, n |
|  | ECG_PAC, n |
|  | ECG_PAC short run, n |
|  | ECG_PAC paired pulses, n |
|  | ECG_PAC frequent occurrence, n |
|  | ECG_SVT, n |
|  | ECG_inferior infarction, n |
|  | ECG_possible inferior infarction, n |
|  | ECG_suspected inferior infarction, n |
|  | ECG_Incomplete right bundle branch block, n |
|  | ECG_indeterminate axis, n |
|  | ECG_combined ventricular hypertrophy, n |
|  | ECG_bifascicular block, n |
|  | ECG_subacute anterior infarction, n |
|  | ECG_subacute anteroseptal infarction, n |
|  | ECG_artificial cardiac pacemaker rhythm, n |
|  | ECG_artificial cardiac pacemaker rhythm.A, n |
|  | ECG_artificial cardiac pacemaker rhythm.D, n |
|  | ECG_low voltage.limb lead, n |
|  | ECG_low voltage.chest lead, n |
|  | ECG_lateral infarction, n |
|  | ECG_possible lateral infarction, n |
|  | ECG_suspected lateral infarction, n |
|  | ECG_coronary vein sinus rhythm, n |
|  | ECG_anteroseptal infarction, n |
|  | ECG_possible anteroseptal infarction, n |
|  | ECG_susptected anteroseptal infarction, n |
|  | ECG_anterior infarction, n |
|  | ECG_possible anterior infarction, n |
|  | ECG_suspected anterior infarction, n |
|  | ECG_counterclockwise, n |
|  | ECG_right ventricular hypertrophy, n |
|  | ECG_right ventricular hypertrophy and right atrial enlargement, n |
|  | ECG_right ventricular hypertrophy and left atrial enlargement, n |
|  | ECG_right atrial enlargement, n |
|  | ECG_dextrocardia, n |
|  | ECG_right axis deviation, n |
|  | ECG_boundary Q wave, n |
|  | ECG_complete right bundle block, n |
|  | ECG_complete left bundle block, n |
|  | ECG_complete atrioventricular block, n |
|  | ECG_left ventricular hypertrophy, n |
|  | ECG_left ventricular hypertrophy and left atrial enlargement, n |
|  | ECG_suspected left ventrical hypertrophy, n |
|  | ECG_left atrial enlargement, n |
|  | ECG_suspected left ventricular rhythm, n |
|  | ECG_left anterior hemiblock, n |
|  | ECG_suspected left anterior hemiblock, n |
|  | ECG_left poterior hemiblock, n |
|  | ECG_left axis deviation, n |
|  | ECG_giant negative T, n |
|  | ECG_flat-low T, n |
|  | ECG_bradycardia, n |
|  | ECG_ventricular trigeminy, n |
|  | ECG_ventricular bigeminy, n |
|  | ECG_intraventricular conduction disorder, n |
|  | ECG_ventricular extrasystole, n |
|  | ECG_venticular extrasystole short run, n |
|  | ECG_ventricular extrasystole double pulse, n |
|  | ECG_ventricular extrasystole, frequent pulse, n |
|  | ECG_ventricular rhythm, n |
|  | ECG_atrial flutter, n |
|  | ECG_atrial fibrillation, n |
|  | ECG_acute inferior infarction, n |
|  | ECG_acute anterior infarction, n |
|  | ECG_suspected acute anterior infarction, n |
|  | ECG_atrioventricular block grade I, n |
|  | ECG_atrioventricular block grade II (Wenckebach), n |
|  | ECG_atrioventricular block grade II (Mobitz II), n |
|  | ECG_atrioventricular block grade II (2:1), n |
|  | ECG_ventricular extrasystole associated with atrioventricular block, n |
|  | ECG_atrioventricular junctional rhythm, n |
|  | ECG_atrioventricular dissociation, n |
|  | ECG_clockwise, n |
|  | ECG_normal range, n |
|  | ECG_sinus arrhythmia, n |
|  | ECG_sinus bradycardia, n |
|  | ECG_sinoatrial block, n |
|  | ECG_sinus tachycardia, n |
|  | ECG_abnormal Q wave, n |
|  | ECG_indeterminate arrhythmia, n |
|  | ECG_escaped beat, n |
|  | ECG_recording faults, n |
|  | ECG_mild right ventricular hypertrophy, n |
|  | ECG_mild left ventricular hypertrophy, n |
|  | ECG_mild left axis deviation, n |
|  | ECG_mild QT prolongation, n |
|  | ECG_mild right ventricular hypertrophy and right atrial enlargement, n |
|  | ECG_mild right ventricular hypertrophy and left atrial enlargement, n |
|  | ECG_mild left ventricular hypertrophy and left atrial enlargement, n |
|  | ECG_suspected mild abnormal ST-T, n |
|  | ECG_mild ST elevation, n |
|  | ECG_mild abnormal ST.T, n |
|  | ECG_negative T, n |
|  | ECG_electrode errot, n |
|  | ECG_tachycardia, n |
|  | ECG_high T wave, n |
|  | ECG_possible elevated posterior infarction, n |
|  | ECG_suspected elevated posterior infarction, n |
|  | ECG_severe right axis deviation, n |
|  | ECG_severe bradycardia, n |
|  | ECG_severe tachycardia, n |
|  | ECG_high voltage, n |
|  | ECG_coved mild ST elevation. right thorax, n |
|  | ECG_coved ST elevation. right thorax, n |
|  | ECG_J wave-associated ST elevation, n |
|  | ECG_P-R shortening, n |
|  | ECG_PR prolongation, n |
|  | ECG_QT prolongation, n |
|  | ECG_QT shortening, n |
|  | ECG_poor R progression, n |
|  | ECG_RSR pattern, n |
|  | ECG_S1 S2 S3 pattern, n |
|  | ECG_abnormal ST-T, n |
|  | ECG_ST elevation, n |
|  | ECG_ST elevation-associated right bundle branch block, n |
|  | ECG_saddleback ST elevation.right thorax, n |
|  | ECG_WPW syndrome, n |
|  | ECG_suspected WPW syndrome, n |
|  | ECG_suspected WPW syndrome A-type, n |
|  | ECG_suspected WPW syndrome B-type, n |
|  | ECG_suspected WPW syndrome C-type, n |
|  | ECG_WPW syndrome A-type, n |
|  | ECG_WPW syndrome B-type, n |
|  | ECG_WPW syndrome C-type, n |
| Computed tomography (CT) | CT_suspected pneumoconiosis = no, n (%)  CT_abnormal cardiac great blood vessel = yes, n (%)  CT_abnormal mediastinum.pulmonary hilum = yes, n (%)  CT_pulmonary mass = yes, n (%)  CT_abnormal pleura.parapet = yes, n (%)  CT_suspected diffusive lung disease = yes, n (%)  CT_bulla, bleb = yes, n (%)  CT_moderate emphysema= yes, n (%)  CT_middle mediastinum pulmonary mass= yes, n (%)  CT_abnormal mammary gland = yes, n (%)  CT_anterior mediastinum pulmonary mass = yes, n (%)  CT_aneurysm = yes, n (%)  CT_suspected primary lung cancer = yes, n (%)  CT_right coronary arterial calcification = yes, n (%)  CT_aortic calcification = yes, n (%)  CT_left anterior descending branch calcification = yes, n (%)  CT_left circumflex branch calcification = yes, n (%)  CT_posterior mediastinum pulmonary mass = yes, n (%)  CT_cardiac dilatation = yes, n (%)  CT_chronic inflammation = yes, n (%)  CT_photographing error = yes, n (%)  CT_tracheal.bronchial stenosis = yes, n (%)  CT_suspected active pulmonary tuberculosis = no, n (%)  CT_atelectasis = yes, n (%)  CT_thyroid mass = yes, n (%)  CT_not particular = yes, n (%)  CT_mediastinum.hilar lymph node calcification = yes, n (%)  CT_mediastinum.hilar adenopathy = yes, n (%)  CT_abnormal pulmonary area = yes, n (%)  CT_pleura plaque = yes, n (%)  CT_pleural callosity.pleurodesis.pleural calcification = yes, n (%)  CT_pleural.chest wall tumor = yes, n (%)  CT_post-thoracoscopy status = yes, n (%)  CT_benign tumor = yes, n (%)  CT_mild emphysema = yes, n (%)  CT_obsolete pulmonary tuberculosis = yes, n (%)  CT_osseous abnormalities = yes, n (%)  CT_severe emphysema = yes, n (%)  hasCT = true (%)  location_right upper lung field.upper lobe, n (%)  location_right lower lung field.lower lobe, n (%)  location_right middle lung field.middle lobe, n (%)  location_left upper lung field.segmentum superius, n (%)  location_left lower lung field.lower lobe, n (%)  location_left middle lung field.lingula, n (%)  location_whole lung field, n (%)  location_pulmonary hilum.mediastinum, n (%) |
| Bundled or calculated from questionnaires | Cough, n |
|  | Phlegm, n |
|  | Abnormal breathing, n |
|  | Allergic symptoms, n |
|  | Has treatment for lung or bronchus, n |
|  | Pack_year, pack/days × years |
|  | Smoking duration, years |
|  | Years from smoking cessation, years |

Abbreviations: ALB, albumin; ALP, alkaline phosphatase; ALT, serum alanine aminotransferase; AST, aspartate aminotransferase; BMI, body mass index; BUN, blood urea nitrogen; CHE, cholinesterase; CRE, creatinine; CRP, C-reactive protein; D-BIL, direct bilirubin; eGFR, estimated glomerular filtration rate; ECG, electrocardiogram; EOS, eosinophil count; FEV_1_, forced expiratory volume in 1 second; FVC, forced vital capacity; GGT, gamma-glutamyl transferase; Hb, hemoglobin; HbA1c, hemoglobin A1c; HDL-C, high-density lipoprotein cholesterol; IRI, insulin; KET, ketone bodies; LDL, low-density lipoprotein cholesterol; MCH, mean corpuscular hemoglobin; MCHC, mean corpuscular hemoglobin concentration; MCV, mean corpuscular volume; PAC, premature atrial contraction; PLT, platelet; RBC, red blood cell; SG, urine specific gravity; SVT, supraventricular tachycardia; T-BIL, total bilirubin; TC, total cholesterol; TP, total protein; UA, uric acid; U-RBC, red blood cell in urine; U-CRE, creatinine in urine; UPH, urine pH; U-WBC, white blood cell in urine; VC, vital capacity; WBC, white blood cell; WPW, Wolf–Parkinson–White.

## Supplementary Table 2. Predictors of logistic regression coefficients

| **Variables** | **Coefficient** |
| --- | --- |
| Arrhythmia | -0.268 |
| Other diseases | -0.288 |
| VC | 0.225 |
| FEV_1_ | -0.229 |
| MCV | -0.383 |
| MCH | 0.571 |
| RBC | n/a^*^ |
| Smoking status | 0.372 |
| Pack-year | n/a^*^ |
| BMI | -0.148 |
| MCHC | -0.123 |
| Cough | 0.072 |
| ALB | -0.013 |
| PLT | 0.163 |
| CRP | -0.043 |
| Smoking duration | -0.518 |
| HT | -0.055 |
| TC | n/a^*^ |
| TP | 0.004 |
| HbA1c | 0.034 |
| %FEV_1_ | 0.836 |
| HDL-C | n/a^*^ |
| AST | -0.079 |
| eGFR | n/a^*^ |
| GGT | n/a^*^ |
| %VC | -0.781 |
| UA | 0.030 |
| ALT | n/a^*^ |
| Duration of smoking cessation | -0.126 |

*Removed by recursive feature elimination in the training logistic regression model

ALB, albumin; ALT, serum alanine aminotransferase; AST, aspartate aminotransferase; BMI, body mass index; CRP, C-reactive protein; eGFR, estimated glomerular filtration rate; FEV_1_, forced expiratory volume in 1 second; GGT, gamma-glutamyltransferase; HbA1c, hemoglobin A1c; HDL-C, high-density lipoprotein cholesterol; HT, hematocrit; MCH, mean corpuscular hemoglobin; MCHC, mean corpuscular hemoglobin concentration; MCV, mean corpuscular volume; PLT, platelet; RBC, red blood cell; TC, total cholesterol; TP, total protein; UA, uric acid; VC, vital capacity

Variables with a positive value for coefficient of logistic regression are indicative of a greater risk of rapid decliner status whereas those with a negative value are unlikely to be predictive of rapid decliner status

## Supplementary Table 3. Predictors of logistic regression coefficients including questionnaire data

| Variables | Coefficient |
| --- | --- |
| Other diseases | -0.243 |
| FVC | 0.511 |
| FEV_1_ | -0.433 |
| MCV | -0.458 |
| Arrhythmia | -0.266 |
| Smoking status | 0.348 |
| Pack-year | 0.208 |
| BMI | -0.077 |
| Physical activity at work | 0.173 |
| RBC | -0.369 |
| MCH | 0.446 |
| MCHC | -0.164 |
| Body fat percentage | -0.045 |
| UPH | 0.075 |
| Regular exercise | -0.206 |
| Age | -0.016 |
| Volume of alcohol consumption per day | 0.274 |
| HDL-C | 0.190 |
| Antilipidemic agents | 0.347 |
| HbA1c | 0.181 |
| PLT | 0.193 |
| HT | 0.137 |
| ALP | -0.071 |
| TC | -0.077 |
| %FEV_1_ | 0.896 |
| Average sleeping time in the last month | -0.275 |
| Duration of smoking cessation | -0.119 |
| Hypertension | 0.278 |
| Having breakfast everyday | 0.225 |
| HB | 0.108 |

ALP, alkaline phosphatase; BMI, body mass index; FEV_1_, forced expiratory volume in 1 second; FVC, forced vital capacity; HbA1c, hemoglobin A1c; HDL-C, high-density lipoprotein cholesterol; HT, hematocrit; MCH, mean corpuscular hemoglobin; MCHC, mean corpuscular hemoglobin concentration; MCV, mean corpuscular volume; PLT, platelet; RBC, red blood cell; TC, total cholesterol; UPH, urine pH

Variables with a positive value for coefficient of logistic regression are indicative of a greater risk of rapid decliner status whereas those with a negative value are unlikely to be predictive of rapid decliner status
